# New York State Lunatic Asylum

**Published:** 1857-09

**Authors:** 


*New York State Lunatic Asylum.*—The late destruction of this spacious edifice by fire is indeed a public calamity. The extraordinary fact that of the hundreds of its patients, all were saved is the only redeeming feature of the startling intelligence. Yet even this result, it would seem, was only attained by the sacrifice of one of its physicians, Dr. Rose, who perished from the injuries he received at the fire. Another martyr to professional fidelity.—*American Med. Gazette.*

